# Characteristics and overall survival of patients with early‐stage non‐small cell lung cancer: A cohort study in Denmark

**DOI:** 10.1002/cam4.4946

**Published:** 2022-06-20

**Authors:** Vera Ehrenstein, Katrine Eriksen, Aliki Taylor, Leslie Servidio, Erik Jakobsen

**Affiliations:** ^1^ Department of Clinical Epidemiology Aarhus University Aarhus Denmark; ^2^ AstraZeneca Cambridge UK; ^3^ AstraZeneca Gaithersburg Maryland USA; ^4^ Department of Thoracic Surgery Odense University Hospital Odense Denmark

**Keywords:** cohort study, epidermal growth factor receptor mutation, non‐small cell lung cancer, overall survival

## Abstract

Non‐small cell lung cancer (NSCLC) accounts for the majority of all lung cancer diagnoses, and approximately 35% of patients with NSCLC are diagnosed at an early stage (I–IIIA). This study aimed to describe epidermal growth factor receptor (*EGFR*) testing, patient characteristics, and overall survival (OS) among patients with early‐stage NSCLC in Denmark. Patients with early‐stage NSCLC registered in the Danish Lung Cancer Registry in 2013–2018 were followed through 2019. We described *EGFR* testing, descriptively summarised patient characteristics, and calculated OS by *EGFR* testing and mutation status. The association between *EGFR* mutation (*EGFR*m) and all‐cause mortality was estimated using Cox proportional‐hazards regression, in subgroups defined by stage at diagnosis, age at diagnosis, comorbidity, and receipt of surgery. In 2013–2018, 21,282 patients with NSCLC were registered in the Danish Lung Cancer Registry, of whom 8758 were diagnosed at an early stage. Of those, 4071 (46%) were tested for *EGFR*m at diagnosis. Median OS was 5.7 years among patients with *EGFR*m‐positive status (*n* = 361) and 4.4 years among patients with *EGFR*m‐negative status (*n* = 3710). *EGFR*m‐positive status was associated with lower all‐cause mortality in all subgroups. This study contributes to population‐based evidence on the epidemiology of early‐stage NSCLC treated in routine clinical practice.

## Graphical abstract

This study describes epidermal growth factor receptor (EGFR) testing and overall survival among patients with early‐stage (I‐IIIA) non‐small cell lung cancer (NSCLC) in Denmark. Patients identified in the Danish Lung Cancer Registry in 2013‐2018 were followed through 2019. Of 8758 patients diagnosed with early‐stage disease, 4071 (46%) were tested for EGFR mutation at diagnosis; EGFR mutation‐positive status was associated with longer overall survival. This study contributes to population‐based evidence on epidemiology of early‐stage NSCLC treated in routine clinical practice.

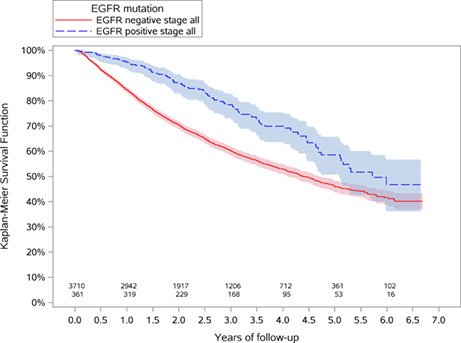

## 1 INTRODUCTION

Non‐small cell lung cancer (NSCLC) accounts for approximately 85% of all lung cancer cases; of those, only approximately 35% are diagnosed at an early stage.^1^, ^2^ Data from 20 countries indicate that five‐year survival ranges between 60% and 90% in stage I NSCLC, and between 12% and 40% in stage III NSCLC.^3^ Advanced and/or metastatic stage disease is associated with even worse survival.^4^ Adjuvant chemotherapy adds a survival benefit of around 5% at 5 years.^5^ Early‐stage diagnosis^3^, ^4^ and early detection^6^, ^7^ are important predictors of survival among patients with NSCLC, and lung cancer screening is efficacious in reducing lung cancer mortality.^8^ Prevalence of epidermal growth factor receptor (*EGFR)* gene mutation in NSCLC is higher in the Asian populations (40%–60%), and lower in the White populations (10%–20%).^5^, ^9^, ^10^, ^11^

The 2017 European Society for Medical Oncology (ESMO) guidelines specify that the primary treatment for potentially resectable early‐stage (I–IIIA) disease is surgery, alone or with adjuvant chemotherapy.^12^ However, following surgery, 45% of patients with stage IB disease and 76% of patients with stage III disease have disease recurrence or die over a median follow‐up of approximately 5 years, regardless of the use of postoperative chemotherapy.^5^ In 2021, ESMO updated its guidelines to recommend adjuvant treatment with the targeted therapy osimertinib for adult patients with resected stage IB–IIIA NSCLC whose tumours have *EGFR* exon 19 deletions or exon 21 L858R substitution mutations.^13^

Data on the prevalence of *EGFR* mutation in early‐stage NSCLC are scarce, as guidelines currently recommend testing only in advanced disease.^14^ *EGFR*‐tyrosine kinase inhibitors (*EGFR*‐TKIs) are the current standard of care for patients with advanced *EGFR* mutant (*EGFR*m) NSCLC, and can prolong survival significantly.^11^, ^15^ As these are driver mutations, their prevalence is assumed at this time to be similar at all stages. This study aimed to describe epidemiology of early‐stage NSCLC in routine clinical practice in Denmark with respect to testing, prevalence of *EGFR*m, demographic and clinical characteristics, initial treatment, and overall survival (OS) according to *EGFR*m, stage, age, comorbidity, and receipt of surgery.

## 2 MATERIALS AND METHODS

This was a cohort study set in Denmark. With its population of 5.8 million and tax‐supported universal health care, Denmark has multiple registries of routinely collected data, including information on inpatient and outpatient hospital diagnoses, migrations, and deaths.^16^ The reporting of new primary malignancies is mandatory. Routine data collection and exact linkage enable complete ascertainment and follow‐up of patients in epidemiologic studies.

Patients with early‐stage (stage I–IIIA) NSCLC registered in the Danish Lung Cancer Registry (DLCR) between 2013 and 2018 were eligible for inclusion in this study. The DLCR is a population‐based nationwide clinical quality database that tracks all patients with incident primary lung cancer diagnosis in Denmark since 2003. The DLCR contains data on age, sex, mutation testing status and results, tumour stage at diagnosis, lung function, performance status, and initial treatment. It links data on comorbidity from the Danish National Patient Registry; on histology from the Danish National Pathology Registry; and on death from the Danish Civil Registration System.^17^

Descriptive statistics of patient characteristics at the time of lung cancer diagnosis were: calendar year of diagnosis, median follow‐up time to death or censoring, stage at diagnosis, sex, age, *EGFR* testing status, mutation status among the tested, forced ejection volume (FEV1), Eastern Cooperative Oncology Group (ECOG) performance status, Charlson Comorbidity Index (CCI) score, smoking history, tumour histology, and initial treatment. OS was defined as time from the date of lung cancer diagnosis to date of death from any cause, with follow‐up censored at emigration, or end of observation, on 10 September 2019. We reported median OS by *EGFR* testing status and, among patients who were tested, by mutation status. Among the patients who were tested, we plotted OS according to *EGFR* mutation status and estimated the association of *EGFR* mutation status with all‐cause mortality in subgroups defined by stage at diagnosis, age at diagnosis, sex, CCI score category, and receipt of surgery. In each subgroup, we estimated hazard ratios (HRs) adjusted for sex, age, calendar year of diagnosis, FEV1, ECOG performance status, and CCI score category. OS was estimated using the Kaplan–Meier method, and adjusted HRs and 95% confidence intervals (CIs) were estimated using Cox proportional‐hazards regression.

To avoid disclosure of personal data and according to Danish data protection regulation, implicit or explicit nonzero cell counts <5 were masked. Implicit cell counts, i.e., those that allowed recomputation of <5 cells from surrounding data, were marked as non‐reportable (NR) regardless of the observed cell count.

## 3 RESULTS

Between 2013 and 2018, we identified 21,282 patients with NSCLC in the DLCR. Data on stage at diagnosis were available for 19,926 (93.6%) patients. There were 8758 (44.0%) patients with early‐stage (I–IIIA) NSCLC at diagnosis, and 4071 (46.5%) of these had been tested for *EGFR* mutation at diagnosis. Table 1 shows the characteristics of eligible patients by testing and mutation status. Most of the patients who were tested for *EGFR* mutation (86.1%) had the most common histological NSCLC subtype of adenocarcinoma,^18^ reflecting that during the study period only patients with adenocarcinoma were routinely tested for *EGFR* mutation. Patients tested for *EGFR* had a higher prevalence of females (57.5% vs. 45.0%), a lower prevalence of FEV1 below 40% predicted (29.1% vs. 37.0%), and a higher prevalence of ECOG performance status of 0 (55.8% vs. 49.9%) than untested patients (Table 1).

**TABLE 1:** Demographic and clinical characteristics of patients with early‐stage (I–IIIA) NSCLC in Denmark, 2013–2018, overall and by *EGFR* testing/mutation status

	*EGFR* testing/mutation status								All patients with stage I–IIIA NSCLC	
Characteristic	Untested		Tested		Negative		Positive			
	*N* = 4687		*N* = 4071		*N* = 3710		*N* = 361		*N* = 8758	
	*N*	%	*N*	%	*N*	%	*N*	%	*N*	%
Sex										
Male	2579	55.0	1730	42.5	1623	43.7	107	29.6	4309	49.2
Female	2108	45.0	2341	57.5	2087	56.3	254	70.4	4449	50.8
Age, years										
<60	548	11.7	615	15.1	563	15.2	52	14.4	1163	13.3
60–69	1369	29.2	1369	33.6	1265	34.1	104	28.8	2738	31.3
70–79	2009	42.9	1560	38.3	1419	38.2	141	39.1	3569	40.8
≥80	761	16.2	527	12.9	463	12.5	64	17.7	1288	14.7
Calendar year of lung cancer diagnosis										
2013	765	16.3	465	11.4	414	11.2	51	14.1	1230	14.0
2014	739	15.8	623	15.3	568	15.3	55	15.2	1362	15.6
2015	746	15.9	687	16.9	635	17.1	52	14.4	1433	16.4
2016	786	16.8	741	18.2	667	18.0	74	20.5	1527	17.4
2017	834	17.8	745	18.3	688	18.5	57	15.8	1579	18.0
2018	817	17.4	810	19.9	738	19.9	72	19.9	1627	18.6
Current or former smoker	3989	85.1	3561	87.5	3259	87.8	302	83.7	7550	86.2
FEV1, % predicted										
<40	1301	37.0	929	29.1	895	30.6	34	12.8	2230	33.2
40–59	1664	47.3	1575	49.4	1433	49.0	142	53.6	3239	48.3
60–79	531	15.1	643	20.2	564	19.3	79	29.8	1174	17.5
≥80	21	0.6	43	1.3	33	1.1	10	3.8	64	1.0
Missing	1170	25.0	881	21.6	785	21.2	96	26.6	2051	23.4
ECOG performance										
0	2122	49.9	2121	55.8	1896	54.6	225	67.8	4243	52.7
1–2	1904	44.8	1547	40.7	1443	41.6	104	31.3	3451	42.8
>2	228	5.4	136	3.6	133	3.8	NR	NR	364	4.5
Missing	433	9.2	267	6.6	238	6.4	29	8.0	700	8.0
CCI score										
Low(0)	1751	37.4	1720	42.3	1535	41.4	185	51.2	3471	39.6
Medium (1, 2)	1919	40.9	1615	39.7	1484	40.0	131	36.3	3534	40.4
High (3+)	1017	21.7	736	18.1	691	18.6	45	12.5	1753	20.0
Histology										
Adenocarcinoma	1166	24.9	3505	86.1	3174	85.6	331	91.7	4671	53.3
Other	3521	75.1	566	13.9	536	14.4	30	8.3	4087	46.7
Initial treatment modality^a^										
Surgery	2739	58.4	2449	60.2	2196	59.2	253	70.1	5188	59.2
Adjuvant therapy	613	13.1	726	17.8	651	17.5	75	20.8	1339	15.3
Radiotherapy	1930	41.2	1796	44.1	1682	45.3	114	31.6	3726	42.5
Chemotherapy	1396	29.8	1562	38.4	1421	38.3	141	39.1	2958	33.8
Neoadjuvant therapy	48	1.0	64	1.6	NR	NR	NR	NR	112	1.3

Abbreviations: CCI, Charlson Comorbidity Index; ECOG, Eastern Cooperative Oncology Group; EGFR, epidermal growth factor receptor; FEV1, forced ejection volume; N, number; NR, non‐reportable; NSCLC, non‐small cell lung cancer.

^a^ Not mutually exclusive.

Of the 4071 patients tested for *EGFR* mutation, 361 (8.9%) were *EGFR*m‐positive, and 3710 (91.1%) were *EGFR*m‐negative. Of the patients with *EGFR*m‐positive status, a higher proportion were female (70.4% vs. 56.3%), a higher proportion had an ECOG performance status of 0 (67.8% vs. 54.6%), a higher proportion had low comorbidity burden (51.2% vs. 41.4%), and a higher proportion received surgery (70.1% vs. 59.2%) compared with patients with *EGFR*m‐negative status. Of note, fewer than half [*n* = 2449; 47.2%] of the 5188 patients who received surgery were tested for *EGFR* mutation. Furthermore, a slightly higher proportion of patients with *EGFR*m‐positive status than patients with *EGFR*m‐negative status received adjuvant therapy (20.8% vs. 17.5%). Age distributions were similar across mutation status groups (Table 1).

**FIGURE 1:**
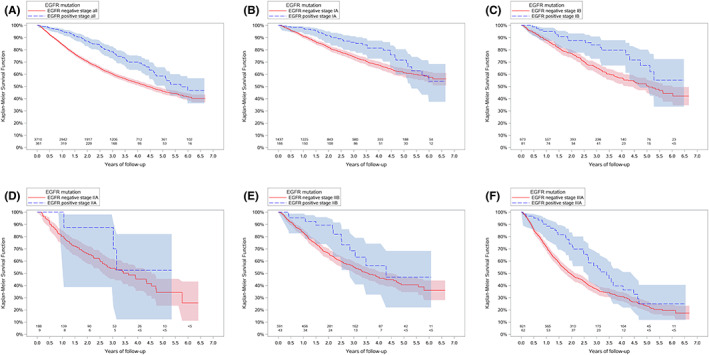
Overall survival and *EGFR* mutation status among patients with early‐stage (I–IIIA) NSCLC, overall and by stage at diagnosis. *The figure shows OS by *EGFR* mutation status overall (Panel A), stage IA (Panel B), stage IB (Panel C), stage IIA (Panel D), stage IIB (Panel E), and stage IIIA (Panel F); EGFR, epidermal growth factor receptor; NSCLC, non‐small cell lung cancer; OS, overall survival.

Among the 4071 patients tested for *EGFR* mutation, median follow‐up time until death or censoring was 2.1 years (IQR: 1.2–3.6 years). Among the 361 patients with *EGFR*m‐positive status, median follow‐up until death or censoring was 2.8 years (IQR: 1.6–4.1 years), and median OS was 5.7 years. Among the 3710 patients with *EGFR*m‐negative status, median follow‐up until death or censoring was 2.1 years (IQR: 1.1–3.5 years), and median OS was 4.4 years. Figure 1 shows Kaplan–Meier curves for OS among patients with early‐stage NSCLC tested for *EGFR* mutation, overall and by stage at diagnosis.

Table 2 shows adjusted HRs for all‐cause mortality by *EGFR*m‐positive versus negative status and according to the subgroups. *EGFR*m‐positive status was associated with a lower all‐cause mortality in all strata of stage at diagnosis, age, sex, comorbidity, and regardless of receiving surgery: adjusted subgroup‐specific hazard ratios for all‐cause mortality associated with *EGFR*m varied from 0.48 to 0.83 (Table 2).

**TABLE 2:** Adjusted hazard ratios for all‐cause mortality over the total follow‐up time among patients with *EGFR*m‐positive versus *EGFR*m‐negative early‐stage NSCLC in Denmark, by stage at diagnosis, age, comorbidity and receipt of surgery

Subgroup	*EGFR*m status	*N* patients	Deaths by end of follow‐up	Adjusted HR (95% CI)^a^
Stage at diagnosis				
IA	Negative	1437	383	Ref
	Positive	166	32	0.83 (0.54–1.29)
IB	Negative	673	242	Ref
	Positive	81	NR	0.59 (0.34–1.03)
IIA	Negative	188	NR	Ref
	Positive	9	NR	0.48 (0.11–2.07)
IIB	Negative	591	NR	Ref
	Positive	43	12	0.59 (0.29–1.21)
IIIA	Negative	821	513	Ref
	Positive	62	33	0.60 (0.38–0.95)
Sex				
Male	Negative	1623	728	Ref
	Positive	107	NR	0.61 (0.38–0.98)
Female	Negative	2087	749	Ref
	Positive	254	66	0.65 (0.48–0.87)
Age group, years				
<60	Negative	563	NR	Ref
	Positive	52	NR	0.75 (0.35–1.57)
60–69	Negative	1265	438	Ref
	Positive	104	NR	0.56 (0.31–1.00)
70–79	Negative	1419	604	Ref
	Positive	141	40	0.67 (0.45–0.99)
≥80	Negative	463	NR	Ref
	Positive	64	25	0.63 (0.39–1.03)
CCI				
Low (0)	Negative	1535	502	Ref
	Positive	185	NR	0.70 (0.48–1.02)
Medium (1, 2)	Negative	1484	605	Ref
	Positive	131	NR	0.55 (0.37–0.83)
High (3+)	Negative	691	370	Ref
	Positive	45	NR	0.74 (0.40–1.38)
Receipt of surgery				
No	Negative	1514	907	Ref
	Positive	108	NR	0.55 (0.38–0.78)
Yes	Negative	2196	570	Ref
	Positive	253	47	0.77 (0.54–1.10)

Abbreviations: CCI, Charlson Comorbidity Index; CI, confidence interval; EGFRm, epidermal growth factor receptor mutation; HR, hazard ratio; N, number; NSCLC, non‐small cell lung cancer; NR, non‐reportable to avoid implicit or explicit reporting of personal data such as counts <5. Implicit counts are those that can be back‐calculated from surrounding data; Ref, reference category.

^a^ Adjusted (unless stratified) for calendar year of diagnosis, age, sex, stage, FEV1, performance status, and comorbidity.

## 4 DISCUSSION

In this population‐based cohort study among patients with early‐stage (I–IIIA) non‐small cell lung cancer in Denmark, nearly half of the patients underwent *EGFR* gene mutation testing at diagnosis of disease, and 8.9% of the tested patients were *EGFR*m‐positive. Patients who were not tested for *EGFR* mutation had worse lung function and performance status than patients who were tested. The presence of a positive *EGFR*m was associated with a lower all‐cause mortality at one and 5 years after lung cancer diagnosis, regardless of disease stage at diagnosis and after adjustment for several prognostic factors such as age, sex, comorbidity, or receipt of surgery.

The prevalence of *EGFR*m in our study population (8.9%) was slightly lower than that reported in systematic reviews for European populations,^11^, ^18^ higher than in an earlier Danish study on a similar population,^19^ and consistent with the prevalence reported in Europe from routine clinical practice.^9^ Overall survival in this study among patients with *EGFR*m‐negative status (the largest group) was comparable to sex‐ and stage‐specific estimates reported recently for a population of patients with NSCLC from Australia, Canada, Denmark, Ireland, New Zealand, Norway, and the UK by Araghi et al (2021).^20^

The advent of precision medicine has led to the availability of treatments for genomic alterations in NSCLC. The SELECT study of adjuvant erlotinib in patients with *EGFR*m‐positive early‐stage (I–IIIA) NSCLC showed an improved 2‐year disease‐free survival (DFS) compared with historic genotype‐matched controls.^21^ The ADJUVANT/CTONG1104 study concluded that adjuvant gefitinib led to longer DFS compared with that for vinorelbine plus cisplatin in patients with completely resected stage II–IIIA *EGFR*‐mutated NSCLC (HR: 0.60; 95% CI: 0.42–0.97).^22^ However, this did not translate to improved OS.^23^ In the Phase III RADIANT study, adjuvant erlotinib did not prolong DFS in patients with stage IB–IIIA *EGFR*‐expressing NSCLC, nor in the subgroup with *EGFR*m‐positive status.^24^ In the ADAURA study of patients with stage IB–IIIA *EGFR*m–positive NSCLC, adjuvant treatment with osimertinib reduced the risk of disease recurrence or death compared with placebo by 83% in patients with stage II and IIIA disease (HR: 0.17; 95% CI: 0.11–0.26), and by 80% in the overall trial population of patients with stage IB–IIIA disease (HR: 0.20; 95% CI: 0.14–0.30); 88% of patients with stage IB disease, were alive and disease‐free at 24 months (HR: 0.39; 95% CI: 0.18–0.76). Adjuvant osimertinib also reduced the risk of central nervous system‐related disease recurrence or death by 82% compared to placebo in patients with stage IB–IIIA disease (HR: 0.18. 95% CI: 0.10–0.33).^15^ Results from these trials led to the approval of osimertinib for the treatment of patients with stage IB–IIIA resected NSCLC by the FDA^25^ and the EMA.^26^ Based on the ADAURA data, there is a need for increased *EGFR* testing in patients with stages I–IIIA NSCLC to identify these patients as early as possible for appropriate adjuvant treatment selection.

Data used in this study were collected for quality assurance, independent of a research question, with data quality likely independent of *EGFR* mutation status, and the DLCR captures the entire eligible population. Limitations of this study include lack of data on cause of death in the analysis dataset; potential unmeasured confounding, and the small number of observations in some subgroups, resulting in low precision of the corresponding estimates.

This study contributes to population‐based evidence regarding selected demographic and clinical characteristics and survival of patients with early‐stage NSCLC treated in routine clinical practice in Denmark. Testing for *EGFR* mutation status in early‐stage disease is recommended given that *EGFR*‐TKI is an approved adjuvant treatment for patients with *EGFR*‐mutated NSCLC. Characterising patients with early‐stage disease, and understanding the impact of *EGFR* mutation in this population, will help guide future treatment decisions.

## AUTHOR CONTRIBUTION

Vera Ehrenstein, Aliki Taylor and Erik Jakobsen provided substantial contribution to the conception and design of the work, drafting of the work and revising it critically for intellectual content. Katrine Eriksen conducted the data analysis and provided substantial contribution to the conception and design of the work, drafting of the work and revising it critically for intellectual content. Leslie Servidio provided substantial contribution to the conception and design of the work, drafting of the work and revising it critically for intellectual content. All authors approved the final version to be published and agree to be accountable for all aspects of the work in ensuring that questions related to the accuracy or integrity of any part of the work are appropriately investigated and resolved.

## FUNDING INFORMATION

This study received institutional research funding from AstraZeneca to and administered by Aarhus University.

## CONFLICT OF INTEREST

Vera Ehrenstein and Katrine Eriksen are salaried employees of Aarhus University or Aarhus University Hospital. Aliki Taylor and Leslie Servidio are or were at the time of the study conduct salaried employees of AstraZeneca. Erik Jakobsen is a salaried employee of Odense University Hospital.

## ETHICS STATEMENT

This study underwent mandatory registration with the Danish Data Protection Agency at Aarhus University (Serial Number 1764) and at the Southern Denmark Region (Journal Number 19/11881). An ethical approval or an approval from an Institutional Review Board is not required for studies based on routinely collected data according to Danish law.

## Data Availability

This study is based on data from the Danish Lung Cancer Registry, which is one of the Danish national databases organised under the Danish Health Data Authority/Danish Ministry of Health. Researchers access data based on all required permission and based on a specific study protocol. Other parties may apply for their own data access, using the standard application/approval process. Further information is available from the corresponding author upon request.
